# Characterization of Pathogenic *Vibrio parahaemolyticus* from the Chesapeake Bay, Maryland

**DOI:** 10.3389/fmicb.2017.02460

**Published:** 2017-12-15

**Authors:** Arlene J. Chen, Nur A. Hasan, Bradd J. Haley, Elisa Taviani, Mitch Tarnowski, Kathy Brohawn, Crystal N. Johnson, Rita R. Colwell, Anwar Huq

**Affiliations:** ^1^Department of Cell Biology and Molecular Genetics, Maryland Pathogen Research Institute, University of Maryland, College Park, College Park, MD, United States; ^2^CosmosID Inc., College Park, MD, United States; ^3^Maryland Department of Natural Resources, Annapolis, MD, United States; ^4^Maryland Department of the Environment, Baltimore, MD, United States; ^5^Department of Environmental Science, Louisiana State University, Baton Rouge, LA, United States; ^6^Maryland Institute for Applied Environmental Health, University of Maryland, College Park, College Park, MD, United States; ^7^Johns Hopkins Bloomberg School of Public Health, Baltimore, MD, United States; ^8^Center for Bioinformatics and Computational Biology, University of Maryland, College Park, College Park, MD, United States

**Keywords:** *Vibrio parahaemolyticus*, Chesapeake Bay, pathogenicity, virulence, environment

## Abstract

*Vibrio parahaemolyticus* is the leading cause of bacterial gastroenteritis associated with seafood consumption in the United States. Here we investigated the presence of virulence factors and genetic diversity of *V. parahaemolyticus* isolated from water, oyster, and sediment samples from the Chesapeake Bay, Maryland. Of more than 2,350 presumptive *Vibrio* collected, more than half were confirmed through PCR as *V. parahaemolyticus*, with 10 encoding both *tdh* and *trh* and 6 encoding only *trh*. Potentially pathogenic *V. parahaemolyticus* were then serotyped with O1:KUT and O3:KUT predominant. Furthermore, pulsed-field gel electrophoresis was performed and the constructed dendrogram displayed high diversity, as did results from multiple-locus VNTR analysis. *Vibrio parahaemolyticus* was readily isolated from Chesapeake Bay waters but was less frequently isolated from oyster and sediment samples collected during this study. Potentially pathogenic *V. parahaemolyticus* was isolated in fewer numbers and the isolates displayed expansive diversity. Although characteristics of the pathogenic *V. parahaemolyticus* were highly variable and the percent of pathogenic *V. parahaemolyticus* detected was low, it is important to note that, pathogenic *V. parahaemolyticus* are present in the Chesapeake Bay, warranting seafood monitoring to minimize risk of disease for the public, and to reduce the economic burden of *V. parahaemolyticus* related illness.

## Introduction

*Vibrio parahaemolyticus*, a halophilic Gram-negative bacterium, is both autochthonous to the marine environment and a causative agent of seafood-related illnesses (Alam et al., 2009). First reported in Japan in the 1950s, *V. parahaemolyticus* has now been recognized as one of the leading causes of seafood-related bacterial gastroenteritis worldwide and accounts for almost 50% of all food poisoning outbreaks in Taiwan, Japan, and Southeast Asia (Martinez-Urtaza et al., 2004; Alam et al., 2009). In the United States, *V. parahaemolyticus* is the leading cause of seafood-induced bacterial enteritis, typically related to consumption of raw or undercooked seafood (DePaola et al., 2003). This pathogen was first identified in 1971 in Maryland, United States after three outbreaks of 425 gastroenteritis cases, in total, were found to be associated with consumption of improperly cooked crabs (Molenda et al., 1972). Subsequently, sporadic outbreaks have occurred throughout the coastal United States (Letchumanan et al., 2014). According to the Centers for Disease Control and Prevention, infection by *V. parahaemolyticus* is estimated to have an annual rate of 4,500 cases per year in the United States (DePaola et al., 2003). The nationwide Cholera and Other Vibrio Illness Surveillance (COVIS) system and Foodborne Diseases Active Surveillance Network (FoodNet) have both reported an increase in vibriosis per 100,000 population from 1996 to 2010 (Newton et al., 2012). Similarly, between 2005 and 2013, there have been 326 reported cases of *Vibrio* related infection in Maryland and of non-cholera related *Vibrio* infections, 38.9% (*n* = 129) were traced to *V. parahaemolyticus* (Agarwal, 2014). Illness caused by *V. parahaemolyticus* can occur 3–24 h after the consumption of contaminated food and symptoms include diarrhea, nausea, vomiting, abdominal cramps, and low-grade fever (Taniguchi et al., 1985). Despite growing understanding of the occurrence and pathogenicity of *V. parahaemolyticus*, the burden of *V. parahaemolyticus* related disease has constantly increased in frequency and range since 2000 (Martinez-Urtaza et al., 2004; Caburlotto et al., 2010). Similarly, in 2013, the USDA estimates that the cost estimate for *V. parahaemolyticus* related disease is 43 million dollars per year (USDA, 2017).

*Vibrio parahaemolyticus* is both oxidative and fermentative and occurs naturally in both marine and freshwater environments where it interacts with various marine and estuarine organisms (Alam et al., 2009; Caburlotto et al., 2010). *Vibrio* species are known to concentrate in the gut of oysters and other filter-feeding bivalves, leading to a higher risk of infection to humans ingesting raw or undercooked seafood (Froelich et al., 2013). Although not the focus of this study, previous studies have detected the occurrence of *V. parahaemolyticus* in various fish species, prawn, and shrimp (Kagiko et al., 2001; Pal and Das, 2010). Prior studies have demonstrated environmental parameters most closely associated with occurrence and distribution of *V. parahaemolyticus* are water temperature and salinity (Kagiko et al., 2001; Caburlotto et al., 2010). When environmental conditions are favorable, increased growth of *Vibrio* species in the water column can lead to increased abundance in filter-feeding bivalves and mollusks. Earlier studies carried out in the Chesapeake Bay region have shown *V. parahaemolyticus* is rarely isolated when the water temperature is below 15°C (Kaneko and Colwell, 1973; Caburlotto et al., 2010). However, it is hypothesized that *Vibrio* species can persist in sediment during colder months, and can then be released back into the water column once temperatures are conducive for growth, usually in the late spring and early summer. Since *V. parahaemolyticus* can persist in estuarine and marine environments year-round, there is a need to determine when the risk of illness, from pathogenic *V. parahaemolyticus*, is highest.

Despite their abundance in estuarine and marine environments, the vast majority of *V. parahaemolyticus* isolated from the environment are not pathogenic, whereas the majority isolated from clinical sources are Shinoda and Miyoshi (2006). The two major and most commonly referenced virulence factors for *V. parahaemolyticus* are thermostable direct hemolysin (*tdh*) and thermostable direct hemolysin-related hemolysin (*trh*) (Nishibuchi and Kaper, 1985; Taniguchi et al., 1986; Kagiko et al., 2001; DePaola et al., 2003; Shinoda and Miyoshi, 2006; Alam et al., 2009; Wang et al., 2015). Both *trh* and *tdh* have similar hemolytic activity *in vitro*, both cause the lysis of human erythrocytes (Wang et al., 2015). The *tdh* gene, which codes for the Kanagawa phenomenon (KP), characterized by β-hemolysis of human erythrocytes, and is typically absent (<1%) in environmental isolates whereas more than 90% of clinical isolates are positive (Martinez-Urtaza et al., 2004; Alam et al., 2009). The KP has been regarded as an important indicator in the identification of the pathogenic and non-pathogenic *V. parahaemolyticus* strains (Wang et al., 2015).

Kanagawa phenomenon negative, clinical *V. parahaemolyticus* isolates were discovered to produce a second hemolysin, *trh*, which unlike *tdh*, is heat labile but immunologically similar to *tdh* (Honda and Iida, 1993). In 1997 in Calcutta, an outbreak of *V. parahaemolyticus* revealed the beginning of a unique serotype, O3:K6, which later became the predominant serotype for *V. parahaemolyticus* related outbreaks (Nair et al., 2007; Ansede-Bermejo et al., 2010). Pandemic O3:K6 strains carry the *tdh* but not the *trh* gene and are generally defined by a positive group-specific PCR (GS-PCR) based on the gene sequences of *toxRS* and opening reading frame, ORF8, from the f237 phage (Matsumoto et al., 2000). The ORF8 of f237 is claimed to be a specific genetic marker of the pandemic isolates of O3:K6 (Nair et al., 2007).

This study characterized a large number of *V. parahaemolyticus* isolates (1,304) collected from sampling sites in the Chesapeake Bay over a 3 year period, focusing on *trh* and/or *tdh* positive strains to determine potential pathogenicity of *V. parahaemolyticus* collected from the Chesapeake Bay, and to determine both genomic relatedness and environmental distribution, if any, of potentially pathogenic *V. parahaemolyticus*.

## Materials and Methods

### Sample Collection

**FIGURE 1:**
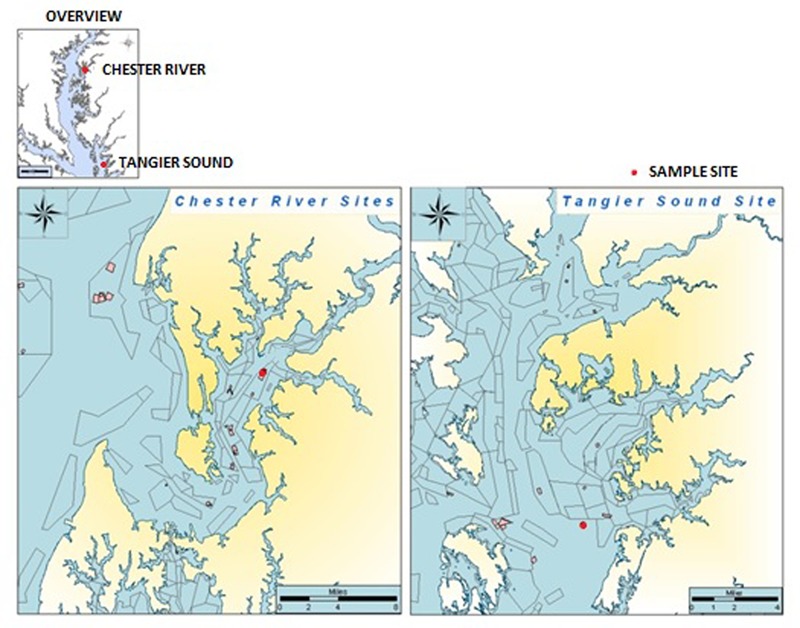
Map of Chesapeake Bay showing sampling sites in the Chester River and Tangier Sound.

Water, oyster, and sediment samples were collected at two locations in the Chester River (39°05.09′N, 76°09.50′W) and Tangier Sound (38°10.97′N, 75°57.90′W) in the Chesapeake Bay, Maryland from June, 2009 to August, 2012 (**Figure 1**). During the warmer months of June through August, sampling was done twice each month and once each month during September through May. At each site, 12 liters of surface water, 20–25 oysters, and 80–100 g of sediment were collected. Oysters were collected by dredging. Samples were kept on ice during transport to the University of Maryland, College Park, MD, United States and, upon arrival, stored overnight at 15°C until processing the following morning.

### Sample Processing

Details of the sample processing have been described elsewhere (Johnson et al., 2010, 2012). Briefly, water samples were shaken and three volumes (1000, 100, and 10 ml), each in triplicate, were resuspended into alkaline peptone water (10X APW, pH 8.5) (111, 11, and 1.1 ml, respectively) and incubated for 16–18 h, with shaking at 30 rpm. Water was not filtered before resuspension with APW. Oysters were rinsed and scrubbed under running water to remove debris stuck to oyster shells, shucked, and the oyster tissue was homogenized 1:1 with 1X phosphate buffer solution (1X PBS; pH 7.4) in a sterile blender for 90 s. Homogenized oyster tissue was inoculated (10 g, 1 g, 0.1 g, in triplicate) into 10X APW and incubated at 33°C for 16–18 h, with shaking at 30 rpm. Sediment samples were weighed and vortexed in equal part 1X PBS, after which 10X APW was added and the samples incubated at 33°C for 16–18 h, with shaking at 30 rpm. The following day, a loopful of pellicle from each overnight samples were collected along with a loopful of shaken overnight samples and streaked individually onto selective media, including CHROMagar^TM^ (CHROMagar, Springfield, NJ, United States) and thiosulfate citrate bile salts sucrose agar, TCBS (Oxoid, Nepean, ON, Canada). The plates were incubated at 37°C for 16–18 h. Presumptive colonies of *V. parahaemolyticus*, based on growth media, were picked and streaked onto LB agar (BD Diagnostic Systems, Sparks, MD, United States) to obtain pure cultures.

### DNA Extraction and PCR

Presumptive isolates of *V. parahaemolyticus* were inoculated into LB broth, incubated at 37°C for 16–18 h with shaking at 150 rpm. A 1.5 ml aliquot of inoculum was centrifuged for 10 min at 13G and the supernatant discarded. To each pellet, 700 μl Tris-EDTA Buffer (TE Buffer; pH 8.0) was added and mixed. Cell suspensions were boiled for 10 min at 99°C, after which the samples were cooled before centrifugation for 10 min at 13G. The supernatant was transferred to a clean, sterile tube and adjusted to concentration for PCR analysis. Multiplex PCR targeting the *toxR* gene (Bauer and Rørvik, 2007) was used to differentiate *V. parahaemolyticus, V. vulnificus*, and *V. cholerae*, and to confirm identification of the isolates. Subsequent PCR targeting virulence factors, *tlh, trh*, and *tdh* (Bej et al., 1999), was done for all confirmed *V. parahaemolyticus* strains. PCRs targeting the group-specific *toxR* variant, GS-PCR, and opening reading frame, ORF8, were performed (Matsumoto et al., 2000). All PCR assays were performed using Promega GoTaq Green Master Mix (Promega, Madison, WI, United States). Each reaction tube contained a total of 25 μl, including 5 μl template DNA. Thermal cycling conditions were as follows: one 10 min cycle of denaturation at 94°C, followed by 36 cycles of denaturation at 94°C for 30 s, annealing temperature for 30 s, extension at 72°C for 60 s, and final extension for 10 min at 72°C. PCR products were stored at 4°C until gel electrophoresis visualization. Sequences, amplicon size and annealing temperatures for each PCR can be found in **Tables 1, 2**. Positive controls included VPTX2103, VPFIHES98, VPAQ41037, and VPF11-3A. Appropriate negative controls were included in all PCR reactions.

**Table 1:** List of primers, annealing temperatures (T_a_), and sequences used to characterize *Vibrio parahaemolyticus* isolates.

Primers	Primer sequence (5′–3′)	Amplicon (bp)	T_a_ (°C)	Reference
utox-F	GASTTTGTTTGGCGYGARCAAGGTT		55	Bauer and Rørvik, 2007
vptox-R	GGTTCAACGATTGCGTCAGAAG	297		
vvtox-R	AACGGAACTTAGACTCCGAC	640		
vctox-R	GGTTAGCAACGATGCGTAAG	435		
tlh-L	AAAGCGGATTATGCAGAAGCACTG	450	58	Bej et al., 1999
tlh-R	GCTACTTTCTAGCATTTTCTCTGC			
tdh-L	GTAAAGGTCTCTGACTTTTGGAC	269		
tdh-R	TGGATAGAACCTTCATCTTCACC			
trh-L	TTGGCTTCGATATTTTCAGTATCT	500		
trh-R	CATAACAAACATATGCCCATTTCCG			
GS-F	TAATGAGGTAGAAACA	651	45	Matsumoto et al., 2000
GS-R	ACGTAACGGGCCTACA			

**Table 2:** Description of *V. parahaemolyticus* VNTR loci and primers used for MLVA.

Locus	Chromosome	Primers	Primer Sequence (5′–3′)	Amplicon (bp)	Motif	Reference
VPTR1	1	VPTR1-F	TAACAACGCAAGCTTGCAACG	255	TATCTC	Kimura et al., 2008
VP2892		VPTR1-R	TCATTCTCGCCACATAACTCAGC			
VPTR2	2	VPTR2-F	GTTACCAAACTGGCGATTACGAAG	615	GCTGTT	Kimura et al., 2008
VPA1454		VPTR2-R	CGGAATTCAGGATCATCCTGAT			
VPTR3	2	VPTR3-F	CGCCAGTAATTCGACTCATGC	333	ATCTGT	Kimura et al., 2008
VPA0714		VPTR3-R	AAGACTGTTCCCGTCGCTGA			
VPTR4	1	VPTR4-F	AAACGTCTCGACATCTGGATCA	229	TGTGTC	Kimura et al., 2008
VP0446		VPTR4-R	TGTTTGGCTATGTAACCGCTCA			
VPTR5	1	VPTR5-F	GCTGGATTGCTGCGAGTAAGA	202	CTCAAA	Kimura et al., 2008
VP3012.VP3013		VPTR5-R	AACTCAAGGGCTGCTTCGG			
VPTR6	1	VPTR6-F	TGTCGATGGTGTTCTGTTCCA	312	GCTCTG	Kimura et al., 2008
VP2226		VPTR6-R	CTTGACTTGCTCGCTCAGGAG			
VPTR7	1	VPTR7-F	CAACAGTTCTGCTCTAATCTTCCG	221	CTGCTC	Kimura et al., 2008
VP2131		VPTR7-R	CAAAGGTGTTACTTGTTCCAGACG			
VPTR8	1	VPTR8-F	ACATCGGCAATGAGCAGTTG	306	CTTCTG	Kimura et al., 2008
VP2956		VPTR8-R	AAGAGGTTGCTGAGCAAGCG			
VP2-07/VPTR16	2	VPTR207-F	ATCGCTGCTTGAAGAAAATCCTGAT	461	TCGTTG	Kimura et al., 2008
VPA1455		VPTR207-R	CTAATTTTTCTGGTTGGGCTTGCG			

### Hemolysis

Cultures of *V. parahaemolyticus* were grown overnight on LB for 18 h at 37°C, streaked onto 5% sheep blood agar, and incubated at 37°C for 18 h. Green hemolysis was defined as α, β as clear hemolysis, and γ as no hemolysis.

### Serotyping

Denken antisera kit containing 13 lipopolysaccharide (O) and 71 capsular (K) sera was used to determine serotypes of pathogenic isolates via slide agglutination. First, *V. parahaemolyticus* isolates were grown overnight at 37°C on 3% NaCl LB agar. Subsequently, a loopful of culture was mixed with 1 ml of 90% normal saline. Half of the cell suspension was boiled at 99°C for 2 h and used for O serotyping whereas the remaining suspension was used for K serotyping. (Denka; Seiken Corp., Tokyo, Japan).

### PFGE

Pulsed-field gel electrophoresis (PFGE) of *V. parahaemolyticus* DNA was performed using a slight modification of the CDC Pulse-Net protocol created by the CDC (PulseNet United States, 2013), as follows.

#### Gel Plug Creation and Lysis

Cultures were grown for 16–18 h at 37°C on LB plates and confirmed for purity. A loopful of each broth culture was mixed with 1 ml cell suspension buffer (CSB) (100 mM Tris: 100 mM EDTA, pH 8). The concentration of cell suspension was adjusted to final absorbance of 0.9 ± 0.1 at 610 nm. Half of the cell suspension was incubated with 25 μl of 20 mg/ml Proteinase K for 10 min at room temperature. Following incubation, 500 μl of cell suspension was mixed with an equal volume of 1% SeaKem Gold agarose pre-warmed to 55–60°C. The solution was transferred to a gel plug mold, dispensed to avoid bubbles, and allowed to solidify for 5 min at 4°C. Each plug was transferred to individual 50 ml Falcon tubes. Each tube contained 5 ml cell lysis buffer (CLB) (50 mM Tris: 50 mM EDTA, 1% sarkosyl, pH 8) and 25 μl Proteinase K (20 mg/ml). Tubes containing plugs, CLBr and Proteinase K were incubated in a 54–55°C water bath with shaking at 150 rpm, for 2 h. Plugs were washed twice with 10 ml sterilized ultrapure water previously warmed to 54–55°C, with shaking, and temperature conditions as above, for 10 min. Additional washes with TE Buffer (10 mM Tris: 1 mM EDTA, pH 8) were performed a minimum of four times. Plugs were stored at 4°C with 5 ml sterile TE buffer until digestion was complete.

#### Digestion and Gel Casting

*Vibrio parahaemolyticus* isolates were SfiI digested and *Salmonella enterica* ATCC BAA-664, serving as control, was XbaI digested. Plugs were cut to 2.0 mm wide slices and inserted into individual 1.5 ml Eppendorf tubes containing pre-digestion master mix consisting of 180 μl sterile ultrapure water and 20 μl 10X restriction buffer per plug. Pre-digestion of *V. parahaemolyticus* was done with incubation at 50°C. *S. enterica* was incubated at 37°C and after 10 min, the pre-digestion buffer was removed and restriction enzyme master mix added. The restriction enzyme master mix for *V. parahaemolyticus* contained 177 μl sterile ultrapure water, 20 μl 10X restriction buffer, 2 μl BSA (10 mg/ml), and 1 μl SfiI (40 U/μl) per plug. The restriction enzyme master mix for *S. enterica* contained 174 μl sterile ultrapure water, 20 μl 10X restriction buffer, 2 μl BSA (10 mg/ml), and 4 μl XbaI (10U/μl) per plug. Plugs were incubated for 4 h at 50°C (*V. parahaemolyticus*) or 37°C (*S. enterica*). Following digestion, the restricted enzyme master mix was removed and 200 μl 0.5X TBE was added to each tube and incubated at room temperature for 5 min. Plugs were loaded onto a gel comb, including control plugs of *S. enterica.* A 1% SeaKem Gold Agarose gel was cast in 0.5X TBE, ensuring plug slices did not move. The agarose gel and plugs were allowed to solidify for at least 30 min and inserted into an electrophoresis chamber containing 4 L freshly prepared 0.5X TBE adjusted to 14°C, with a flow rate of 1 L/min.

#### CHEF Mapper and Staining

The CHEF Mapper electrophoresis chamber program was set to Auto Algorithm, with a low MW of 78 kb and high MW of 396 kb. After running for 18–19 h, the gel was stained in ethidium bromide (10 mg/ml) and visualized.

#### Dendrogram Preparation

Restriction patterns were analyzed using BioNumerics software (Applied Maths, Sint-Martens-Latem, Belgium). The background was subtracted and the normalized before fingerprint patterns were typed.

### DNA Extraction for Sequencing

*Vibrio parahaemolyticus* isolates were grown overnight in LB broth at 37°C for 16–18 h, with shaking at 150 rpm. A 1.5 ml aliquot of inoculum was centrifuged for 10 min at 13G and the supernatant discarded. DNA was extracted using a Qiagen MiniPrep kit, following the manufacturer’s protocol (Qiagen, Venlo, Limburg).

### MLVA

Multiple-locus variable nucleotide tandem repeat (MLVA) analysis was performed for 16 of the *tdh+, trh+ V. parahaemolyticus* strains, employing nine primer sets belonging to both chromosomes 1 and 2. PCR conditions were identical to those described for conventional PCR. After confirmation by PCR, 25 μl PCR product was purified using DNA Clean and Concentrator^TM^-5 (ZymoResearch, Irvine, CA, United States) and mailed for sequencing (Eurofins MWG Operon, Louisville, KY, United States). After sequencing, the number of repeat motifs were counted for each isolate at each individual loci. Primers and repeat motifs for each loci can be found in **Table 2**.

## Results

Water, oyster, and sediment samples collected at sampling stations located in Tangier Sound and the Chester River in the Chesapeake Bay, between June, 2009 and August, 2012, yielded 2,350 presumptive *Vibrio* isolates, of which 1,304 were confirmed *V. parahaemolyticus* by *toxR* targeted multiplex PCR. The remaining isolates were mainly *V. vulnificus* and *V. cholerae.* All 1,304 *V. parahaemolyticus* isolates possessed the species-specific *tlh* gene. Of all *V. parahaemolyticus* isolates, 16 (1.2%) were potentially pathogenic, 10 of which (62.7%) contained both of the virulence encoding genes, *tdh* and *trh.* Six isolates (37.5%) were positive for *trh.* The majority of the Chesapeake Bay *V. parahaemolyticus* strains (83.2%) were isolated from water (whole water, plankton free water, plankton and water), followed by oyster (9.1%), and sediment (7.7%). Of the 16 potentially pathogenic *V. parahaemolyticus*, none were isolated from oyster, assumed because of limitations associated with relying on culture based methods.

The majority of presumptively pathogenic *V. parahaemolyticus* strains collected from Tangier Sound were isolated during the colder months of September, December, and January, 2009–2011. In contrast, the presumptively pathogenic *V. parahaemolyticus* were isolated from the Chester River during the warmer months of May, June, and August, 2009–2010, except for one strain in September, 2010 and two in December, 2009.

Serotyping was performed on all potentially pathogenic *V. parahaemolyticus* strains and the majority contained O1 antigen, followed by O3 and then O5. Most strains could not be typed for the K antigen using conventional kits and the most frequently occurring serotype was O1:KUT, a serovariant of O3:K6, accounted for 37.5% of strains tested, followed by O3:KUT (18.75%) (**Table 3**).

**Table 3:** Characterization of sixteen water and sediment *V. parahaemolyticus* isolates collected from the Chester River and Tangier Sound, Maryland.

Strain ID	Area of isolation	Date of isolation (M/D/Y)	Source	Serotype	Hemolysis	*tlh*	*tdh*	*trh*	GS	ORF8
TR013-02	Tangier Sound	12/15/09	Water	O1:KUT	β	+	+	+	-	-
TS013-07	Tangier Sound	12/15/09	Water	O1:KUT	β	+	+	+	-	-
CR015-02	Chester River	12/07/09	Water	O1:KUT	β	+	+	+	-	-
CR015-09	Chester River	12/07/09	Water	O3:KUT	β	+	+	+	-	-
TS014-10	Tangier Sound	01/21/10	Sediment	O5:K30	β	+	-	+	-	-
TS014-11	Tangier Sound	01/21/10	Sediment	O5:K3	β	+	+	+	-	-
CR021-01	Chester River	05/24/10	Water	O10:KUT	β	+	+	+	-	-
CR021-06	Chester River	05/24/10	Water	O1:KUT	β	+	+	+	-	-
CR022-06	Chester River	06/14/10	Water	O1:KUT	β	+	+	+	-	-
CR022-08B	Chester River	06/14/10	Water	O1:KUT	β	+	+	+	-	-
CR022-14	Chester River	06/14/10	Water	O1:K68	β	+	+	+	-	-
CR026-19A	Chester River	08/16/10	Water	O1:K58	β	+	-	+	-	-
CR028-01	Chester River	09/13/10	Water	O1:K56	β	+	-	+	-	-
TS026-22	Tangier Sound	09/21/10	Water	O3:KUT	β	+	-	+	-	-
TS026-23	Tangier Sound	09/21/10	Water	O3:KUT	β	+	-	+	-	-
TS026-30	Tangier Sound	09/21/10	Water	O3:K59	β	+	-	+	-	-

**FIGURE 2:**
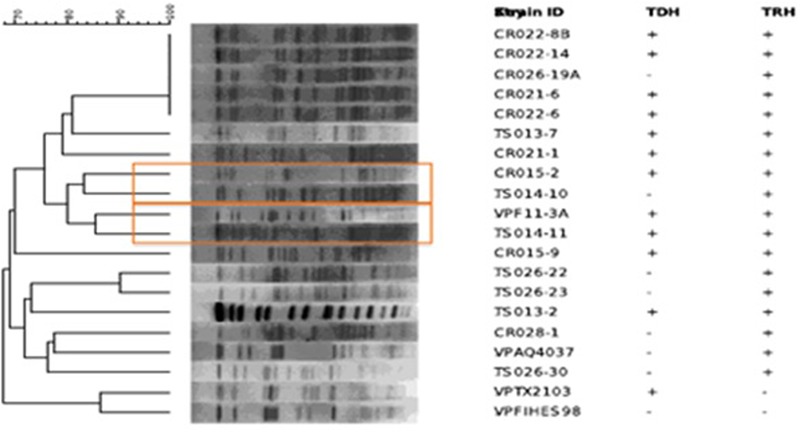
Dendrogram created with Bionumerics showing pulsed-field gel electrophoresis (PFGE) patterns of *Sfi* digested *Vibrio parahaemolyticus* isolates.

Pulsed-field gel electrophoresis patterns of the 16 potentially pathogenic *V. parahaemolyticus* showed significant diversity, five falling into a cluster of related strains, but none showing similar banding patterns (**Figure 2**). None of the strains shared similar MLVA patterns, confirming the diversity detected by PFGE (**Tables 2, 4**).

**Table 4:** Number of tandem repeats present in 16 *trh* and/or *tdh* positive *V. parahaemolyticus* isolates and four reference strains included in the study.

Strain	VPTR1	VPTR2	VPTR3	VPTR4	VPTR5	VPTR6	VPTR7	VPTR8	VPTR207
VPAQ41037	10	22	5	3	7	21	4	10	0
VPF11-3A	9	21	3	3	9	12	4	5	0
VPTX2103	23	14	6	5	7	17	4	8	0
VPFIHES98	16	24	5	6	5	12	4	9	0
TR013-02	20	18	3	1	11	9	4	0	0
TS013-07	5	34	4	2	2	10	5	7	0
CR015-02	1	19	1	0	5	7	4	10	0
CR015-09	10	31	6	2	1	11	4	8	0
TS014-10	4	19	2	3	5	7	4	6	0
TS014-11	9	35	0	0	3	19	4	6	0
CR021-01	12	24	5	1	3	6	4	7	0
CR021-06	12	0	5	2	5	15	4	7	0
CR022-06	12	44	0	1	5	16	4	7	0
CR022-08B	11	34	5	0	5	15	4	7	0
CR022-14	12	46	5	1	5	11	4	7	0
CR026-19A	10	35	2	1	2	8	4	18	0
CR028-01	19	18	5	7	3	20	4	6	0
TS026-22	17	13	5	3	2	19	4	7	0
TS026-23	17	5	5	3	3	18	4	6	0
TS026-30	17	19	5	3	3	18	4	6	0

## Discussion

During the course of this study, *V. parahaemolyticus* was collected from both locations from all sample types in large numbers. However, out of all *V. parahaemolyticus* strains characterized for pathogenicity, based on the presence of either *trh* or *tdh*, less than 2% were found to be potentially pathogenic. In a study performed by Parveen et al. (2008), by comparable enrichment techniques, all samples were negative for both *trh* and *tdh* positive *V. parahaemolyticus*. However, when real time PCR was performed on the same samples collected by Parveen et al. (2008), detection of *tdh* and *trh* positive *V. parahaemolyticus* increased to 20 and 40%, respectively, for oyster samples and 13 and 40% for water samples. Conversely, in a study performed by Davis et al. (2017), water samples collected from the Chesapeake Bay between 2007 and 2010, were all negative for both *trh* and *tdh* during the course of the whole study. Ultimately, the ability to detect pathogenic *V. parahaemolyticus* is greatly impacted by sample processing techniques.

Similar to previous studies performed in the United States (DePaola et al., 2003), in this study, all 16 *tdh+* and/or *trh+* were negative for pandemic markers GS and ORF-8 by PCR, indicating *V. parahaemolyticus* isolates collected during this study are different from pandemic O3:K6 strains (**Table 3**). *V. parahaemolyticus* strains negative for GS-PCR are also negative for ORF-8, the marker for the filamentous phage, presumed associated with pandemic genotypes O3:K6 (Bhuiyan et al., 2002; Alam et al., 2009). The majority of the Chesapeake Bay *V. parahaemolyticus* strains (83.2%) were isolated from water (whole water, plankton free water, plankton and water), followed by oyster (9.1%) and sediment (7.7%). Of the 16 potentially pathogenic *V. parahaemolyticus*, none were isolated from oyster, assumed because of limitations associated with relying on culture based methods and the ability of *V. parahaemolyticus* to undergo a VBNC state (viable but non-culturable) (Mizunoe et al., 2000).

The majority of presumptively pathogenic *V. parahaemolyticus* strains collected from Tangier Sound were isolated during the colder months of September, December, and January, 2009–2011. In contrast, the presumptively pathogenic *V. parahaemolyticus* were isolated from the Chester River during the warmer months of May, June, and August, 2009–2010, except for one strain in September, 2010 and two in December, 2009 (**Table 3**). Interestingly, all presumptively pathogenic *V. parahaemolyticus* were isolated during the first year of the study, with the last of the isolates collected in September 2010, suggesting environmental factors determining temporal changes in the occurrence of these strains of *V. parahaemolyticus*. However, more importantly and likely, is the fact that environmental strains of pathogenic *V. parahaemolyticus* are very difficult to isolate (Mizunoe et al., 2000). In a similar study conducted in India, pathogenic *V. parahaemolyticus* were isolated from 59% of samples after enrichment for 18 h, but the same samples yielded strains negative for *tdh* when conventional methods followed by PCR were employed (Deepanjali et al., 2005). A study carried out in Japan found 41.5% seawater and 8.5% organic matter samples were positive for *tdh* and *trh* when MPN followed by PCR was done but the *tdh* and/or *trh* positive strains could not be isolated (Alam et al., 2003). Similarly, in a study performed in the Chesapeake Bay, detection of *tdh* positive *V. parahaemolyticus*, was not detected in water samples via direct plating and 55% of the time using enrichment methods (Parveen et al., 2008). Thus, it is concluded that potentially pathogenic strains of *V. parahaemolyticus* are present in the Chesapeake Bay, but isolation and culture of these strains can remain a challenge.

*Vibrio parahaemolyticus* associated with disease outbreaks is multi-serogroup, with at least 13 O and 71 K serogroups having been reported (Alam et al., 2009). Commercial kits manufactured in Japan are commonly used to distinguish serogroups (Martinez-Urtaza et al., 2004) and the one most frequently isolated from clinical cases is O3:K6, shown to be the causative agent of a massive outbreak of diarrhea cases in Kolkata, India, in 1996, and later identified in other parts of the world, including Asia, Africa, Europe, Latin America, and the United States (Nair et al., 2007). Results of previous studies have shown that the *V. parahaemolyticus* O3:K6 serogroup contains the O3:K6 specific filamentous phage f237 and GS sequences of the toxRS operon in addition to ORF-8. These are used as markers to distinguish O3:K6 from other serogroups (Alam et al., 2009). Serotypes O1:KUT, O1:K25, O1:K41, and O4:K68 have been shown to be serovariants of O3:K6 (Martinez-Urtaza et al., 2004). The majority of potentially pathogenic *V. parahaemolyticus* strains contained O1 antigen, followed by O3 and then O5. Most strains could not be typed for the K antigen using conventional kits and the most frequently occurring serotype was O1:KUT, a serovariant of O3:K6, accounted for 37.5% of strains tested, followed by O3:KUT (18.75%).

In addition to serotyping, a variety of fingerprinting techniques, including PFGE and MLVA, have been used to profile *V. parahaemolyticus.* Although PFGE is not a new method, few studies have employed PFGE to analyze the diversity of environmental isolates of *V. parahaemolyticus*, especially with respect to geographic distribution. Previous studies employing PFGE have been done in Japan, Bangladesh, Taiwan, and China (Wong et al., 1996; Suffredini et al., 2011; Wang et al., 2017). Only recently have environmental strains of *V. parahaemolyticus* from more than one European country been characterized using PFGE (Suffredini et al., 2011). Furthermore, very few *V. parahaemolyticus* isolates have been in the United States, and specifically within the Chesapeake Bay have been subjected to PFGE analysis, only one study in Texas utilized PFGE analysis for *V. parahaemolyticus* diversity (Daniels et al., 2000). In this study, a dendrogram constructed using PFGE patterns showed significant diversity among the 16 strains of *V. parahaemolyticus* isolated in this study, a conclusion also drawn from results of MLVA (**Figure 2**). Of the 16 potentially pathogenic *V. parahaemolyticus* environmental strains typed by PFGE, none had identical banding patterns, not surprisingly given past studies showing high genetic diversity among *V. parahaemolyticus* strains (Alam et al., 2003, 2009). The lack of duplicate banding patterns amongst these strains is important as PFGE is used to determine the ancestry of bacterial strains. Of the 16 strains, only five fell into a cluster of related strains. Interestingly, those five strains had been isolated from the Chester River on three separate days (the first on May 24, 2010, three on June 14, 2010, and the fifth on August 16, 2010). Given the high diversity among all Chesapeake Bay *V. parahaemolyticus* isolates observed in this study, it is intriguing to note that these five strains formed a related cluster despite having been isolated over a 4 month period. Of the five strains, four were *tdh+* and *trh+* and the strain last to be isolated, on June 14, 2010, was *tdh-*, an interesting observation since significant strain divergence was observed among those carrying *trh* compared to the *tdh+* strains. MLVA is a fingerprinting technique to distinguish bacterial strains with little to no genetic variation (Lüdeke et al., 2015). The amplification of polymorphisms are determined in several Variable-Number Tandem-Repeat (VNTR) loci. These VNTRs are highly polymorphic and can be used to differentiate bacterial strains based on the length of repeat regions. None of the *V. parahaemolyticus* isolates in this study shared similar MLVA patterns, confirming the diversity detected by PFGE (**Tables 2, 4**). Repeats in the VPTR207 locus were not detected in any of the strains and the least variability of repeat regions was observed at locus VPTR7 of chromosome 1, with most strains carrying four or five repeats. Ultimately, strains of potentially pathogenic *V. parahaemolyticus* are extremely diverse in regard to location, time and sample type.

Although the occurrence of *trh* and/or *tdh V. parahaemolyticus* isolates were relatively uncommon, there are other putative virulence factors that could cause pathogenicity. Previous studies have shown that environmental isolates of *V. parahaemolyticus* that lacking *tdh* and/or *trh* were able to produce putative virulence factors, such as extracellular proteases, biofilm, siderophore, and remained cytotoxic (Mahoney et al., 2010). Ultimately, cytotoxicity and enterotoxicity of pathogenic *V. parahaemolyticus* cannot be entirely explained by *tdh* and *trh*, indicating unknown virulence factor may play a role in pathogenicity (Raghunath, 2014).

Ideally, monitoring *Vibrio* species in water, sediment, and oysters should provide a good estimate of the actual occurrence of pathogenic *V. parahaemolyticus* relative to total *Vibrio* spp., if sufficient sampling is done. However, the requirement for an intensive monitoring regimen, coupled with the difficulty in isolating pathogenic *V. parahaemolyticus*, and related pathogens, cause environmental surveillance to remain a serious challenge. However, once patterns for the presence of pathogenic *V. parahaemolyticus* in relation to various environmental parameters, such as temperatures and salinity, are coupled, an effective monitoring program can be provided to guard the public from *Vibrio* related disease and infection. Not only can active monitoring of *Vibrio* safeguard the public from disease and infection, ultimately, monitoring of *Vibrio* can have economic benefits, as USDA estimates that *V. parahaemolyticus* related disease cost an estimated 23 million dollars per year (USDA, 2017).

In summary, Chesapeake Bay strains of *V. parahaemolyticus* can carry indicators of pathogenicity and are highly diverse, however, they represent a low proportion of the total population of *V. parahaemolyticus* in the Chesapeake Bay. These conclusions are in concordance with those reported globally. However, because potentially pathogenic *V. parahaemolyticus* can be readily isolated from the Chesapeake Bay waters, a monitoring program that include *V. parahaemolyticus* would be a wise public health program to help reduce the incidence of *V. parahaemolyticus* related illness.

## Author Contributions

AC, NH, BH, ET, MT, and KB were involved in sample collection, processing and/or lab experiments. AH and RC designed the study in the Chesapeake Bay and supervised the project while CJ was a collaborator in the larger project. All authors reviewed and edited the manuscript written by AC.

## Conflict of Interest Statement

The authors declare that the research was conducted in the absence of any commercial or financial relationships that could be construed as a potential conflict of interest.
